# Association of painful human immunodeficiency virus distal sensory polyneuropathy with aberrant expectation of pain relief: functional magnetic resonance imaging evidence

**DOI:** 10.1093/braincomms/fcab260

**Published:** 2021-10-30

**Authors:** Irina A Strigo, John R Keltner, Ronald J Ellis, Alan N Simmons

**Affiliations:** 1 Emotion and Pain Laboratory Research, San Francisco Veterans Affairs Health Care Center, San Francisco, CA 94121, USA; 2 Department of Psychiatry, University of California San Francisco, San Francisco, CA 94143, USA; 3 Stress and Neuroimaging Laboratory Research, San Diego Veterans Affairs Health Care Center, San Diego, CA 92151, USA; 4 Department of Psychiatry, University of California San Diego, La Jolla, CA 92093, USA; 5 Department of Neurosciences, University of California San Diego, La Jolla, CA 92093, USA; 6 Center of Excellence in Stress and Mental Health Research, San Diego Veterans Affairs Health Care Center, San Diego, CA 92161, USA

**Keywords:** neuropathy, imaging, insula, reward, striatum

## Abstract

Mechanisms underlying chronic neuropathic pain associated with HIV-associated distal sensory polyneuropathy are poorly understood, yet 40% of those with distal neuropathy (or 20% of all people with HIV) suffer from this debilitating condition. Central pain processing mechanisms are thought to contribute to the development of HIV neuropathic pain, yet studies investigating central mechanisms for HIV neuropathic pain are few. Considering the motivational nature of pain, we aimed to examine the degree to which expectation of pain onset and expectation of pain offset are altered in sixty-one male patients with HIV-related distal sensory polyneuropathy with (*N* = 30) and without (*N* = 31) chronic neuropathic pain. By contrasting painful (foot) and non-painful (hand) sites between those with and without neuropathic pain, we could identify unique neural structures that showed altered activation during expectation of pain offset or relief. Our results showed no evidence for peripheral mechanisms evidenced by lack of significant between group differences in thermo-sensation, subjective pain response or epidermal nerve fibre density. Likewise, we found no significant differences between groups in subjective or brain mechanisms underlying the expectation of pain onset. Conversely, we found significant interaction within right anterior insula during expectation of pain offset in our study in that individuals in the pain group compared to the no-pain group exhibited increased anterior insula activation on the painful compared to the non-painful site. Our findings are consistent with abnormal processing of expectation of pain offset or abnormal pain relief-related mechanisms potentially due to increased emotional distress regarding the experience of chronic endogenous pain.

## Teaser

Mechanisms underlying chronic neuropathic pain associated with HIV-associated distal sensory polyneuropathy are poorly understood. Using functional MRI, Strigo et al. show abnormal expectation of pain offset response in those who developed neuropathic pain potentially due to lack of pain relief regarding the experience of chronic endogenous pain.

## Graphical Abstract

**Graphical Abstract:**
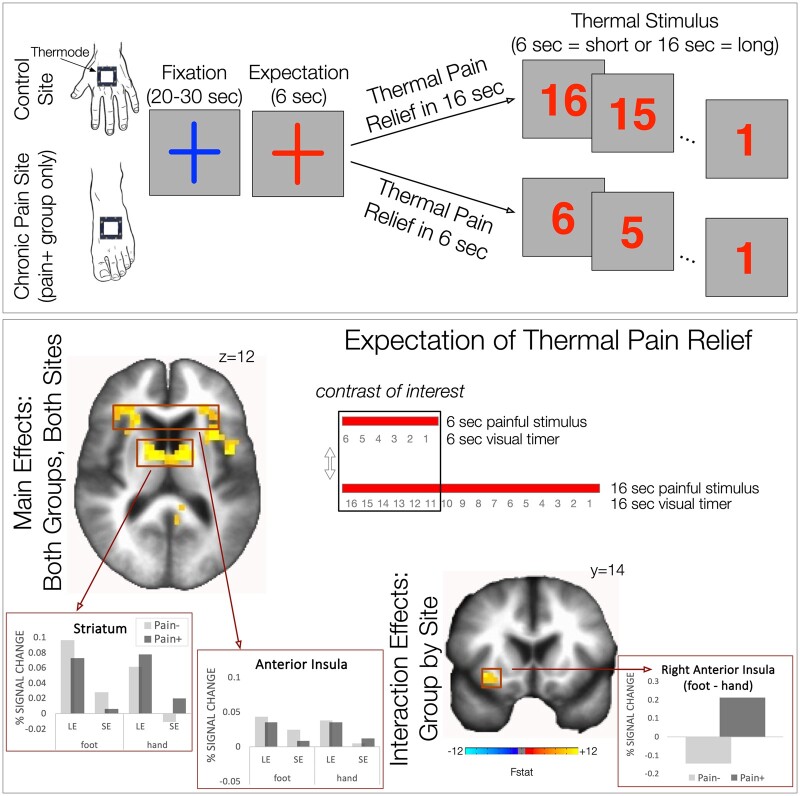

## Introduction

HIV-associated distal sensory polyneuropathy (DSP) is the most prevalent neurologic complication of HIV-1 infection in the era of combination antiretroviral therapy,^1^^,^^2^ affecting 50% of all HIV patients.^3^ It is unclear why 40% of those with distal neuropathy [or 20% of people with HIV] develop chronic neuropathic pain (i.e. pain persisting on a daily basis for three months or more). This clinical syndrome is treatment-resistant and is associated with impaired daily function, unemployment, decreased quality of life and depression.^3^ Additionally, neuropathic pain does not remit with successful virological suppression on antiretroviral therapy and its prevalence is increasing, rather than decreasing.^4^ A better understanding of brain mechanisms for pain processing of HIV distal neuropathic pain (DNP) may help determine why some patients develop chronic pain and others do not. Most HIV neuropathic pain research to date has focused on the role of peripheral mechanisms of nerve injury and sensitization. This work has failed to reveal a mechanistic pathway that fully explains the wide variability of clinical expression of DNP in people with HIV.^5^

In addition to the contribution of peripheral mechanisms, central brain pain processing may also contribute to the development of chronic HIV-DNP. However, CNS pathophysiology associated with HIV peripheral neuropathic pain is not well studied. Recent published research suggests that brain mechanisms contribute to HIV peripheral neuropathy symptoms.^6^ HIV-DNP has been associated with smaller total cerebral cortical grey matter volumes^7^ of total cerebral cortical grey matter and smaller posterior cingulate cortex volumes.^8^

Expectation plays an important role in the perception of pain.^9^ In fact, expectation can be a determinant of how much pain a patient experiences.^10^^,^^11^ Expectation can be so powerful that it can be used as a treatment intervention in the management of pain; this is referred to as placebo analgesia.^12^ Conversely, expectation itself causes pain to be experienced as more severe; this is referred as nocebo hyperalgesia.^13–15^ Nocebo hyperalgesia can be so strong that it interferes with or worsens pain treatment outcomes.^16–21^ Understanding the neurobiology of expectation and its influence on pain transmission offers a path to improving clinical care in people with pain.^9^

It has been known for the past decade that the anterior insula (AI) is a key part of the brain which participates in negative expectation of impending pain.^22–25^ The right AI has been shown to mediate negative valence emotions;^25–28^ in particular, the right AI mediates negative expectation of nocebo hyperalgesia,^13^^,^^15^^,^^29^ increased negative emotional response to experimental pain processing and anticipation in anxiety and depression and emotional allodynia.^30–33^ Here, we hypothesized that a brain mechanism leading to the experience of HIV-DNP may be increased emotional distress in anticipation of pain relief, a process that would translate into a reduced behaviour and increased avoidance in the clinic. We designed a neuroimaging experiment using both pain predicting and pain-relieving cues in order to evaluate the degree to which HIV-DNP disrupts neural processes underlying expectations of pain onset and pain offset.

## Materials and methods 

### Participants

Sixty-one male people with HIV with DSP gave written informed consent to participate in this study, which was approved by the University of California San Diego Human Research Protection Program. All participants were community-dwelling adult volunteers participating in research studies at the HIV Neurobehavioral Research Program at UC San Diego. All 61 participants complained of sensory disturbances in their feet, characterized by loss of sensation, dysesthesia and paresthesia. Thirty out of 61 complained of feet pain and had a diagnosis of DNP at study entry. HIV-DNP was defined as a specific pattern of bilateral burning, aching, or shooting pain in a distal gradient in the lower extremities, as described previously.^3^^,^^34^ Individuals in the DNP group complained of pain in both feet, had signs of neuropathy (specifically, bilateral distal reduction in reflexes, vibration sensation or sharp sensation in the feet on examination by a trained clinician) and additionally reported numbness and tingling consistent with peripheral neuropathy. The groups did not differ significantly on age (*t* = 1.09, *P* = 0.27), education (*t* = 0.03, *P* = 0.96) or race (chi = 3.01, *P* = 0.39) and all were male (see Table 1 for details).

**Table 1:** Participants characteristics

	CNL		DNP		Stats
	Mean	SD	Mean	SD	*t*/*Χ*^2^(pval)
Demographic variables					
Age (years)	58.3	8.3	58.4	7.0	0.03 (0.96)
Education (years)	15.2	3.4	14.3	2.9	1.09 (0.27)
Race					3.01 (0.39)
African American	4		3		
Hispanic	4		1		
Caucasian	23		25		
Other	0		1		

CNL = patients without neuropathic pain; DNP = patients with neuropathic pain; SD = standard deviation.

Potential participants were excluded based on the presence of a neurocognitive morbidity that is external to HIV illness, serious co-morbid medical condition unrelated to HIV, neurological confounds (e.g. head injury with loss of consciousness for greater than 30 min, seizure disorders, CNS neoplasm’s unrelated to HIV, MS), severe psychiatric disorder, current intoxication or active abuse/dependence within last 30 days (based on Composite International Diagnostic Interview, see below). In addition, participants were excluded if they had contraindications to MRI scanning such as pregnancy/breastfeeding, claustrophobia, or metal prosthesis or device. All participants provided written informed consent prior to enrolment and data were collected in accordance with all ethical standards as stipulated by the UC San Diego institutional review board-approved procedures,

### Clinical measures

All participants completed comprehensive Neuromedical assessment that followed standardized HIV Neurobehavioral Research Program protocol that included clinical neurological examination^3^ and psychiatric evaluation using Composite International Diagnostic Interview.^35^ In addition, participants completed a battery of questionnaires assessing specific pain and co-morbid symptoms. The battery included Brief Pain Inventory (BPI),^36^ Gracely Pain Scale,^37^ McGill Pain Questionnaire (MPQ),^38^ Beck Depression Inventory-2 (BDI-2),^39^ Positive and Negative Affect Scale (PANAS),^40^ Profile Of Mood States (POMS),^41^ Pain Catastrophizing Scale (PCS),^42^ Fear of Pain Questionnaire (FOPQ)^43^ and Medical Outcomes Study-HIV Quality of Life.^44^

In addition, every subject underwent skin biopsies at the ankle in order to evaluate for epidermal nerve fibre density (ENFD).^45^ Standard definitions of abnormal HIV peripheral neuropathy ratings have been defined for both individual peripheral neuropathy measures^46^ (ENFD) and summary peripheral neuropathy measures (i.e. the Total Neuropathy Score^47^).

### Temperature sensitivity

The method of constant stimuli was used to measure subjects’ sensitivity to experimental heat stimuli. Heat stimulation started from a baseline of 32°C and rose linearly at a rate of 1.5°/C to one of six predetermined temperatures (44, 45, 46, 47, 47.5, 48°C). The duration of each stimulus was 6 s, excluding the rise/fall time. A 9 cm^2^ thermode (Medoc TSA-II, Ramat-Yishai, Israel) was applied to each subjects’ left foot (painful site for the DNP group) and left hand, and the site of stimulation on the skin was varied slightly to avoid sensitization. The skin under the thermode was adapted to the baseline thermode temperature before the start of stimulation. The interval between successive stimuli was at least 30 s, and the minimum interval between stimulation of the same skin site was at least 1 min. Subjects were asked to rate the pain intensity and unpleasantness of each stimulus using two validated visual scales.^48^ After each temperature stimulus, subjects were asked to rate the maximum sensation of pain using a scale that ranged from 0 (‘no pain sensation’) to 10 (‘extremely intense pain sensation’). Furthermore, subjects rated the maximum unpleasantness evoked by each temperature stimulus, using a scale that ranged from 0 (‘not at all unpleasant’) to 10 (‘extremely unpleasant’).

### Experimental paradigm

**Figure 1:**
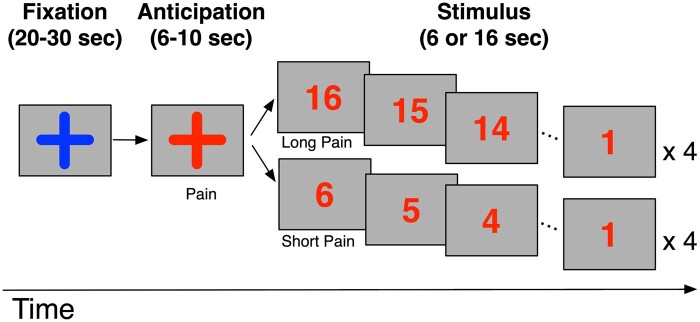
**Experimental paradigm.** Experimental paradigm of expectation of pain onset and pain offset.

We designed an experiment to measure negative expectation both before and during an experimental pain stimulus. This experiment was motivated by our hypothesis that a key brain mechanism for HIV-PNP is negative expectation of pain due to lack of pain relief processes associated with chronic endogenous pain. For this purpose, we designed a novel task that measured, both expectation of pain onset, as well as expectation of pain offset or expectation of pain relief by explicitly cuing participants to impending protracted pain (during the actual pain stimulus, Fig. 1). The negative expectation of protracted pain was designed to better emulate expectation of pain relief processes that seem particularly abnormal in chronic pain patients.^49^

The experimental pain stimulus was a painful thermal heat stimulus applied to the dorsal region of the left hand or dorsal region of the left foot, in semi-randomized order. Temperature stimulus was always applied to the left side, since neuropathy was bilateral in all subjects. The temperature of the pain stimulus was the same on both sites and was chosen from a range of six different temperatures (44–48°C) which the patient identified to be rated 6–7/10 intensity of thermal heat pain prior to scanning (see above). The experimental painful thermal heat stimulus in the scanner was delivered as either a 6 s experimental painful thermal stimulus or a 16 s experimental thermal stimulus. In the scanner, during each temperature stimulus the patient was shown a timer on the screen, which counted down either 6 s for the 6 s stimulus or counted down 16 s for the 16 s stimulus to cue participants to the offset of painful stimulus.

### Imaging data acquisition

Imaging data were acquired on a GE Discovery MR750 3 T whole-body system with a body transmit coil and an 8-channel receive-only head coil at the University of California San Diego Center for Functional MRI. The structural brain sequence consisted of a high-resolution T_1_-weighted Fast Spoiled Gradient Recall (3D FSPGR) scan: 172 1.2 mm contiguous sagittal slices, field of view = 240 mm, repetition time = 8 ms, excitation time = 3.1 ms, flip angle = 8, TI = 600 ms, 256 × 192 matrix. Two 6 min and 36 s functional scans were acquired using a T2*-weighted echo planar image sequence (matrix = 64 × 64; 30 axial slices; in-plane resolution = 3.75 × 3.75 × 4.00; repetition time = 1.5 s; excitation time = 30; flip-angle = 80°).

### Statistical analysis

#### Imaging data processing

Imaging pre-processing was conducted using a combination of Analysis of Functional NeuroImages (AFNI) software package (Cox, 1996). A multivariate regressor approach detailed below was used to relate changes in echo planar image intensity to differences in task characteristics. echo planar image images were co-registered, using an AFNI program that optimally controls for movement. Data were processed through ‘afni_proc.py’ for maximal replicability. Specifically, data were (i) despiked by removal of regional statistical outliers with interpolated regional means, (ii) corrected to slice acquisition, (iii) corrected for six direction motion parameters (*x*, *y*, *z*, roll, pitch, yaw) and their derivatives, (iv) aligned to anatomical T_1_ and normalized brain space, and (v) scaled for percent signal change. Pain task data were regressed using a REsidual Maximum Likelihood model (in AFNI’s ‘3dREML’) with task-based haemodynamics of interest including: (i) anticipation block (6 s), (ii) 6 s pain stimulus block, and (iii) initial 6 s of the 16 s pain stimulus block, in addition to noise regressors including a baseline and linear regressor as well as the six motion parameters (*x*, *y*, *z*, roll, pitch, yaw). The contrasts of interests included (i) expectation of pain onset and (ii) expectation of pain offset (or expectation of pain relief) (note that only the initial 6 s of the pain stimulus of the 16 s stimulus block was included).

Haemodynamics of the pain experience were modelled using line interpolation (3dDeconvolve/3dREMLfit modelled with TENT) for the span from the initiation of stimulus cue and the stimulus [32 s (using 11 TENTs) for the short pain and 48 s (using 17 TENTs) for the long pain stimuli]. These regressors were reconstructed to form a time series with 11 data points 1.5 s apart, which was used in subsequent analysis.

Group differences in expectation of pain onset and expectation of pain offset (or expectation of pain relief) were tested with two separate voxel-wise linear mixed effects models. For the expectation of pain onset, Group (DNP, CNTL) and Location (foot, hand) were entered as fixed factors and subject entered as a random factor with order of stimulation (i.e. hand 1st or foot 1st) entered as a covariate. Note that expectation of 6 s versus 16 s stimulus onset was not differentiated in this model since subjects were not aware of the duration of the upcoming painful stimulus, hence expectation of pain onset was treated as a single event irrespective of the duration of the following stimulus. For the expectation of pain offset or expectation of pain relief model, Group (DNP, CNTL), Exp Pain Relief (6 s, 16 s), Location (foot, hand) were entered as fixed factors and subject entered as a random factor with order of stimulation (i.e. hand 1st or foot 1st) entered as covariate. Analysis was done with the AFNI function 3d linear mixed effects, which uses statistical program R^50^ (www.cran-r.org) and the nlme library. In each linear mixed effects, results were examined for significant main effects of Expectation of Pain Onset and Expectation of Pain Offset (or expectation of pain relief), as well as for Group by Expectation Pain Onset/Pain Offset interaction providing information on the between group differences in expectation of pain onset and offset (or relief) across both stimulation sites. We particularly focused on the Group by Expectation Onset/Offset by Location interaction, which provided information on the between group differences in expectation of pain onset and offset (or relief) while controlling for the non-painful neuropathy site. For all analyses, a voxelwise threshold of *P* < 0.005 was set within the whole brain. Cluster-size thresholds corrected for multiple comparisons at *P* < 0.05 were calculated with AFNI’s 3dClustSim procedure, as recommended^51^^,^^52^ using model parameters for the spatial autocorrelation of the data within the brain. For all clusters surviving the clustersize threshold, we calculated the cluster *F*-values by averaging the voxel-based *F*-values in each cluster. Finally, the average percent signal change was extracted from regions of activation for visualization.

#### *Post hoc* correlations

In order to examine whether expectation of pain offset was related to impact of neuropathic pain in our study, we conducted *posthoc* correlations of voxel-based activation for expectation of pain offset (covarying for age) with pain interference ratings (from BPI) in the pain-only group. This outcome was chosen, as functional interference might be clinically more meaningful and better capture the functional consequences of a chronic pain state.^53^ These findings cluster corrected (voxel = 0.005; cluster = 0.05).

#### Subgroup characterization analysis

*T*-tests and chi-square tests were used to compare subgroups on clinical (i.e. peripheral neuropathy severity, DNP, non-neuropathic pain, paresthesia, dysesthesia, depression, anxiety) and demographic variables (i.e. age, sex, education). Repeated measures ANOVAs were used to compare temperature sensitivity between the two groups. Results were considered significant at *P* < 0.05 (corrected).

### Data availability

The data that support the findings of this study are available on request from the authors.

## Results

### Clinical and psychological variables

Clinical presentation was consistent with DSP in both groups as indicated by bilateral distal reduction in reflexes, vibration sensation or sharp sensation in the feet. Reduction in reflexes, vibration and sharp sensation were not significantly different between the two groups (Table 2). Individuals in the pain group showed increased severity of dysesthesia and paresthesia, as well as increased sensation loss, resulting in higher total neuropathy score of 12.2 (SD = 3.2) versus 6.5 (SD = 2) in the no-pain group (*P* < 0.001). Conversely, skin biopsy data showed that groups did not differ in ENFD measures (*P* > 0.05, Table 2). As expected, subjects in the DNP group exhibited significantly higher levels of pain on all pain assessments (see Table 2). In addition, ratings on the Medical Outcome Survey (MOS) were also significantly worse in the DNP compare to CNL group for both, physical health and mental health summary (*P*’s < 0.05). Those with pain also demonstrated significantly higher symptoms of depression as measured by the BDI-2 (Table 2) yet no significant difference in the total mood disturbance measured by Profile of Mood States (*P* > 0.05). Conversely, ratings of fear of pain or pain catastrophizing, as well as ratings of positive and negative affect were not significantly different between pain and no-pain groups (*P*’s > 0.05, Table 2).

**Table 2:** Clinical and psychological characteristics

	CNL	DNP	*P*-val	*T*-val
Clinician assessed neuropathic pain				
Vibration	0.9 (0.3)	1.0 (0.2)	0.33	0.99
Sharps	0.7 (0.5)	0.7 (0.5)	0.94	0.08
Reflexes	0.7 (0.4)	0.9 (0.3)	0.23	1.22
Dysesthesias: Severity	0.0 (0.0)	2.6 (1.2)	0.00	11.60
Paresthasias: Severity	1.4 (0.8)	1.9 (0.8)	0.01	2.64
Loss of sensation: Severity	1.4 (0.9)	2.8 (1.2)	0.00	5.42
Total neuropathy score	6.5 (2.0)	12.2 (3.2)	0.00	8.30
Skin biopsy				
Epidermal Nerve Functional Density (ENFD)	10 (6.8)	7.9 (7.7)	0.23	1.25
Fear of Pain Questionnaire				
Severe	33.4 (9.6)	31.1 (10.8)	0.37	0.90
Minimal	19.9 (6.7)	18.0 (7.3)	0.30	1.04
Medical	23.6 (7.6)	21.9 (8.0)	0.38	0.89
Total fear of pain	76.9 (19.8)	70.9 (22.4)	0.27	1.11
Pain Catastrophizing Scale (PCS)				
Rumination	6.0 (3.9)	7.5 (3.9)	0.13	1.53
Magnification	3.0 (2.2)	3.2 (2.6)	0.83	0.22
Helplessness	5.2 (4.0)	7.0 (4.9)	0.11	1.61
Total PCS	14.2 (8.7)	17.7 (10.1)	0.15	1.45
Brief Pain Inventory (BPI)				
BPI average (neuropathic pain)	0.0 (0.0)	3.6 (2.2)	0.00	9.29
BPI interference (neuropathic pain)	0.0 (0.0)	3.2 (2.5)	0.00	7.22
BPI average (non-neuropathic pain)	1.6 (2.5)	2.8 (2.3)	0.06	1.89
BPI NNP interference (non-neuropathic pain)	0.8 (1.2)	2.5 (2.7)	0.002	3.25
Medical Outcome Survey (MOS)				
MOS physical health summary	46.1 (8.4)	39.3 (10.0)	0.01	2.91
MOS mental health summary	52.8 (9.0)	46.5 (10.0)	0.01	2.62
Positive and Negative Affect (PANAS)				
PANAS now positive affect	33.0 (7.6)	30.2 (8.1)	0.17	1.38
PANAS now negative affect	12.0 (3.5)	13.1 (5.2)	0.34	0.97
PANAS past year positive affect	32.8 (7.3)	29.5 (9.4)	0.13	1.55
PANAS past year negative affect	16.8 (7.8)	19.3 (9.0)	0.26	1.14
Profiles of Mood States (POMS)				
Total mood disturbance	44.4 (29.4)	61.7 (35.7)	0.06	1.93
Beck Depression Inventory 2 (BDI 2)				
Total depression severity	8.4 (7.9)	15 (10.2)	0.01	2.86
Gracely neuropathic pain now (state)	0.0 (0.0)	8.7 (5.4)	0.0	9.01

CNL = patients without neuropathic pain; DNP = patients with neuropathic pain; Scores format: Mean (standard deviation).

### Temperature sensitivity prior and during scanning

**Figure 2:**
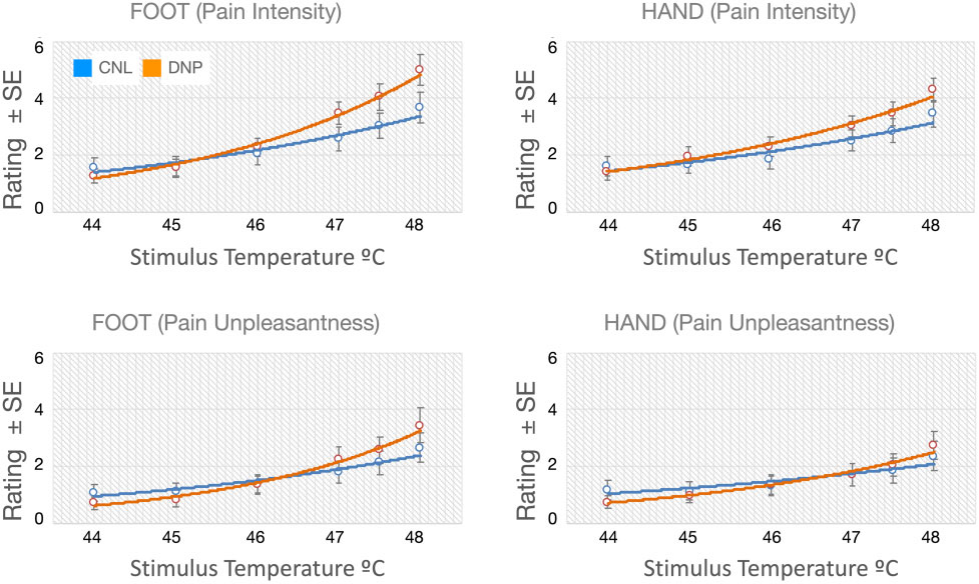
**Psychometric functions. Intensity (*top*) and unpleasantness (*bottom*) ratings to temperature stimuli in subjects without (CNL, blue) and with distal neuropathic pain (DNP, orange).** Participants received six temperature stimulations to their foot (painful site for DNP group) and hand outside the scanner and provided ratings of pain intensity and pain unpleasantness for each stimulation. Both groups provided comparable ratings to temperature stimuli. Repeated measures ANOVA showed no significant effect of group or group by temperature level interactions (*P*’s >0.05). Error bars reflect standard errors (SE).

All subjects received six temperature stimulations to their foot (painful site for DNP group) and hand outside the scanner to compare temperature sensitivity between the groups and determine temperature level for use in the MRI scanner. Subjects’ ratings of both intensity and unpleasantness to these temperature stimuli are depicted in Fig. 2. Both groups provided comparable ratings to temperature stimuli. Repeated measures ANOVA showed no significant effect of group or group by temperature level interactions (*P*’s >0.05). Likewise, there were no between-group differences in temperature intensities used in the MRI scanner [CNL: 47.6 (SD = 0.5)°C; DNP: 47.4 (SD = 0.5)°C, *P* = 0.245; *t* = 1.174]. Finally, post-scan ratings of temperature stimuli during scanning were also comparable between the two groups (Table 3).

**Table 3:** Post-scan participants ratings

Mean (SD)	CNL	DNP	*T*-val^a^	*P*-val
Foot				
Anticipation	2.4 (2.6)	2.4 (2.3)	0.007	0.995
Short pain intensity	3.1 (2.7)	4.0 (2.2)	1.227	0.225
Long pain intensity	6.3 (2.7)	7.3 (2.5)	1.261	0.212
Short unpleasantness	2.4 (2.5)	2.9 (2.2)	0.429	0.670
Long unpleasantness	5.8 (2.9)	6.1 (3.0)	0.319	0.751
Hand				
Anticipation	1.5 (2.4)	1.9 (2.2)	0.612	0.510
Short pain intensity	3.2 (2.3)	3.7 (2.0)	0.703	0.485
Long pain intensity	6.1 (2.3)	6.4 (2.2)	0.428	0.670
Short unpleasantness	2.2 (2.3)	2.9 (2.1)	0.901	0.371
Long unpleasantness	5.1 (2.9)	5.3 (2.4)	0.274	0.785

CNL = patients without neuropathic pain; DNP = patients with neuropathic pain; SD = standard deviation.

a Between group *t*-test.

### Blood oxygen level dependent activation

#### Linear mixed effects expectation of pain onset

No significant clusters were observed for the Group or Group by Location interaction.

#### Linear mixed effects expectation of pain offset

**Figure 3:**
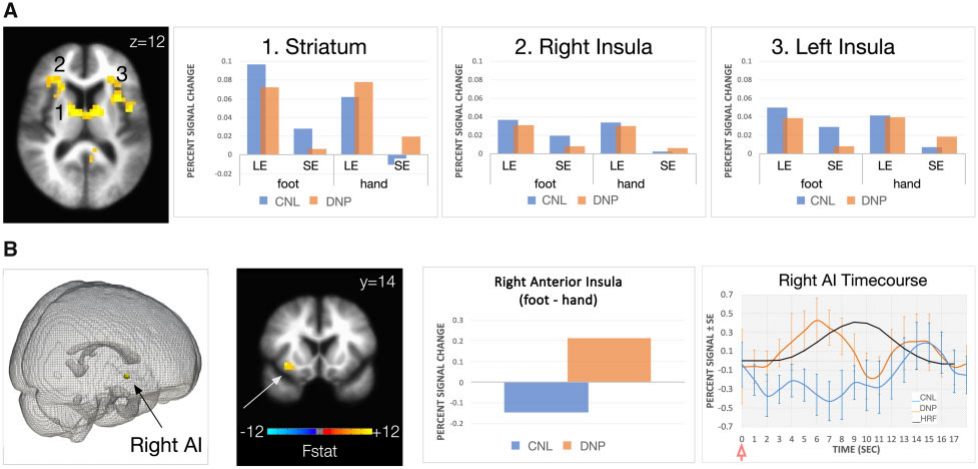
**Main effects and Interactions for expectation of pain offset.** (**A**) Significant main effects of expectation of pain offset were observed in several cortical and subcortical regions, including bilateral anterior insula, striatum (shown), as well as brainstem, and several loci within parietal and occipital lobes (c.f. Table  4 for details) with higher activation during long compared to short temperature stimulus, as expected. Percent signal change for long/short hand/foot stimulation is depicted in the bar graphs for striatum and bilateral insulae. (**B**) Significant Group × Expectation × Location interaction during expectation of pain offset (or pain relief) in the right anterior insula activation. Closer analysis of this interaction showed increased activation within this region during foot (painful site for the DNP group) compared to hand (non-painful site for the DNP group) stimulation for the long compared to short temperature stimulation in the DNP group, as depicted by the percent signal change bar graph. The inset on the bottom right shows results of the TENT function analysis, which depicts increased and shifted activation during the expectation of pain offset on the painful site (foot) compared to the non-painful site (hand) in the pain group (DNP). In other words, anterior insula activation peaks earlier that the predicted hemodynamic response (HRF, bottom right). Red arrow indicates onset of temperature stimulation. CNL = control group; DNP = distal neuropathic pain group; LE = expectation of pain offset during long temperature stimulus (16 s); SE, expectation of pain offset during short temperature stimulus (6 s).

Main effects of expectation of pain offset (or expectation of pain relief) are shown in Fig. 3 and Table 4. Main effects of expectation of pain offset were observed in several cortical and subcortical regions, including bilateral AI, striatum, brainstem, and several loci within parietal and occipital lobes (see Table 4) with higher activation during long compared to short temperature stimulus, as expected. We observed no significant clusters in the Group or Group × Expectation of pain offset interaction. However, the right AI activation showed significant Group × Expectation × Location interaction during expectation of pain offset (or pain relief). Closer analysis of this interaction showed increased activation within this region during foot (painful site for the DNP group) compared to hand (non-painful site for the DNP group) stimulation for the long compared to short temperature stimulation in the DNP group (Fig. 3, bottom, middle). A TENT function analysis showed increased and shifted activation during the expectation of pain offset on the painful site (foot) compared to the non-painful site (hand) in the pain group, in that AI activation peaks earlier that the predicted hemodynamic response (see Fig. 3, bottom right).

**Table 4:** Brain activation: Expectation of pain offset

Brain region (BA)	Side	Volume	Talairach coordinates			Fstat
		mm^3^	*X*	*Y*	*Z*	
Expectation of pain relief: Main effect (Both Groups, Both Locations)						
Anterior insula	Right	5312	27	17	20	10.30
Anterior insula	Left	5184	−33	12	14	10.28
Cingulate (posterior)	Left	11072	−14	−38	48	10.50
Cingulate (posterior)	Right	5696	14	−42	45	10.33
Striatum	Left	4608	−1	−1	12	11.07
Parietal lobe (BA40)	Left	2880	−47	−35	30	12.19
Parietal lobe (BA 40)	Right	2816	50	−35	29	10.33
Occipital lobe (BA18)	Right	14848	24	−71	−11	12.19
Occipital lobe (BA 19)	Left	11968	−26	−72	−13	11.16
Brainstem	Left	3200	−2	−39	4	9.81
Expectation of pain relief: Group × Location interaction						
Anterior insula	Right	512	30	14	−6	9.90

BA = Brodmann’s Area.

### *Post hoc* voxel-based correlations

**Figure 4:**
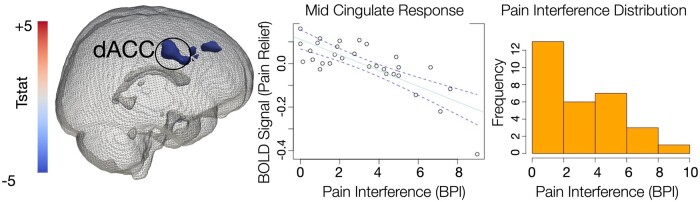
**Voxel-based correlations between pain relief-related brain response and neuropathic pain interference.** Voxel-based correlations between expectation of pain offset activation and levels of pain interference reported by the DNP group (from BPI interference score) covarying for age were conducted in the whole brain in the DNP group only. Activation cluster corrected (voxel = 0.005; cluster = 0.05). Significant inverse correlations were found within anterior mid cingulate (aMCC: *X*/*Y*/*Z* = 1/11/36, volume = 9984 mm^3^) (scatter plot, Pearson correlation between percent signal change and interference scores, 95% confidence intervals) and dorsolateral prefrontal cortex (dlPFC: *X*/*Y*/*Z* = 32/26/25, volume 7552 mm^3^) (scatter plot not shown), suggesting that less activation within these regions was related to greater reported interference of neuropathic pain in these individuals. The inset on the bottom right show distribution of interference scores from BPI in the DNP group only.

To further strengthen aberrant central relief-related processing in the pain group, we performed voxel-based correlations between expectation of pain offset activation and levels of pain interference reported by the DNP group (from BPI interference score). Significant inverse correlations were found within dorsal cingulate and dorsolateral prefrontal cortex, suggesting that less activation within these regions was related to greater reported interference of neuropathic pain in these individuals (Fig. 4).

## Discussion

The aim of this work was to examine brain mechanisms underlying expectation of onset and offset of thermal painful stimuli in a sample of males with HIV-related DSP. We examined the hypothesis that brain mechanism leading to the experience of HIV-DSP induced neuropathic pain may be due to abnormal processing of expectation of pain offset or abnormal expectation of pain relief mechanisms due to increased negative expectation and emotional distress regarding the experience of chronic endogenous pain. Several important findings were observed. First, we found no evidence for peripheral mechanisms evidenced by lack of significant between group differences in thermosensation, nociception or ENFD. Second, we found no significant differences between groups in subjective or brain mechanisms underlying the expectation of pain onset. Third, we found significant interaction within right AI during expectation of pain offset in our study in that individuals in the pain group compared to the no-pain group exhibited increased AI activation on the painful compared to the non-painful site. Our findings are in line with findings underlying abnormal central relief-related processing in other chronic pain conditions (e.g. Baliki et al.^49^) and provide potential mechanism for why some HIV DSP patients experience DNP and others do not.

Our main finding was increased right AI response to expectation of pain offset in the DNP compared to the no-pain group during stimulation of the painful (foot) compared to the non-painful (hand) site. Main effects of expectation of pain offset (or pain relief), which we evaluated by manipulating the duration of the painful stimulus and explicitly cuing participants to the end of pain, were observed within bilateral anterior insular cortex and striatum, as expected. AI is an interoceptive hub that has been strongly linked to pain and emotional processing.^25^ Increased AI activation during aversive processing and anticipation is often related to increased distress and/or anxiety.^54^ Increased AI during negative anticipation of experimental pain, especially in people prone to anxiety or having negative association with the upcoming stimuli is commonly observed.^30^^,^^33^^,^^55^ Less control over impending threat is associated with increased AI response, especially in those with enhanced anxiety.^56^ We found no significant group or group by location differences in anticipation of pain onset in our study. We posit that the lack of differences was due to the fact that participants were not cued to the time of pain onset and thus did not associate this pain onset cue with their endogenous pain. Our findings showed that it was not that pain in general was perceived as more negative by the pain group, rather it was the expectation of pain offset on the painful site that was associated with the increased AI in our study, suggesting that the neuropathic pain group most likely did not associate the end of the painful stimulus on their painful site with pain relief. Our correlational analyses further support this notion, where we found a significant inverse relationship between relief-related mid anterior cingulate activation and participants’ reports of pain interference in the pain positive group. In other words, those with lowest anterior cingulate cortex activation during expectation of pain relief also reported highest pain interference scores in our study. Pain interference can be considered an experimental proxy to pain suffering and lack of pain relief, as it describes consequences of chronic pain on one’s life. Human studies show that the offset of pain (or pain relief) is associated with positive activity change in the rostral and dorsal parts of the anterior cingulate and ventral striatum in humans,^57^^,^^58^ a circuitry that is implicated in pain relief mechanisms in animals.^59^ Furthermore, mid anterior cingulate plays a fundamental role in pain processing as interoceptive motor cortex^60^ by regulating subjective feelings of pain unpleasantness.^61^ Evidence suggests that during (and before) the painful stimulus, the anterior cingulate cortex engages lower parts of the descending pain control system (i.e. periaqueductal grey, hypothalamus, rostral ventromedial medulla), which in turn exert an opioid-dependent inhibitory influence on spinal nociceptive processing, reducing nociceptive input to thalamic and cortical regions, and ultimately leading to a reduced pain experience.^62^ We observed no significant activation in the brainstem in relation to relief-related processes in our study. In turn, our observation is more consistent with the proposed role of the anterior mid cingulate cortex in the adaptive control over pain and punishment while anticipating the offset of pain.^63^^,^^64^ Taken together, our findings support abnormal relief-related processing in those with neuropathic pain associated with HIV-DSP. This neurobiological mechanism may potentially underlie reduced behaviour and increased pain avoidance leading to increased disability in those with chronic pain observed in the clinic. It is widely accepted that the avoidance behaviour in chronic pain patients ultimately results in physical deconditioning, depression, disability from work and an inability to participate in recreation or family activities.^65^ There is substantial evidence that in patients living with chronic pain, avoidance is closely related to increased pain, physical disability and long-term sick leave.^66^ Avoidance responders with chronic pain appear the most burdened, dysfunctional patient group concerning measures of stress, action control, maladaptive coping and health.^67^ Here, we provide a mechanism of how lack of pain relief may perpetuate the avoidance of activities that people with chronic pain associate with the occurrence or exacerbation of pain.

To our knowledge, no prior study has examined experimental pain processing in HIV-DSP induced neuropathic pain. Work in diabetic polyneuropathy strongly points to subcortical, brainstem-mediated modulatory mechanisms involved in the development of neuropathic pain.^68^ Our findings are consistent, as we found significant between-group difference in the expectation of pain relief aspects rather than differences in nociceptive processing. Examination of resting state functional connectivity in HIV-DNP (in submission) shows altered connectivity between the default mode and salience networks as potential mechanisms underlying the development and/or maintenance of HIV-DNP, potentially revealing different manifestations of the default mode network modulatory response to pain. In painful diabetic neuropathy resting state network connectivity is likewise altered.^69–71^ Structural brain abnormalities are also consistently observed in neuropathic pain. In our prior work we showed that more severe DNP was associated with smaller volumes of total cerebral cortical grey matter in HIV-infected individuals even after statistically controlling for several HIV disease-related factors and non-HIV characteristics (e.g. substance abuse and depression).^7^ DNP was not associated with altered subcortical volumes in our prior work,^7^ and smaller midbrain and thalamic volumes were associated with paresthesia rather than pain, while atrophy in the posterior cingulate cortex was related to both pain and paresthesia.^6^^,^^8^ Our prior work did not find consistent evidence for insular or cingulate atrophy in HIV-DNP, suggesting that functional reorganization is more likely.

Our results strongly point to a difference in central pain processing of pain relief due to HIV-DNP. First, we found no clear differences between the pain and no-pain group in thermosensation or nociception, in that subjective ratings for painful and non-painful temperatures did not significantly differ between the two groups. Likewise, temperatures used in the MRI scanner to induce comparable sensations did not differ between the pain and non-pain groups. In other words, thermal sensitivity evaluated by psychophysical sensory thresholds or subjective ratings of suprathreshold stimuli was comparable between the pain and no-pain group in our study. Additionally, our skin biopsies data showed no significant differences between pain and no-pain group in ENFD, further supporting the role of central pain processing in manifestation of neuropathic pain following HIV-DSP. Most HIV neuropathic pain research to date has focused on the role of peripheral mechanisms of nerve injury and sensitization, direct effects of HIV or antiretroviral drugs on peripheral nerves (e.g. exposure to dideoxynucleoside reverse transcriptase inhibitors such as stavudine or didanosine) and on clinical risk factors for neuropathy (age, height and lower CD4 nadir).^3^ This work has failed to reveal a mechanistic pathway that fully explains the wide variability of clinical expression of DNP in HIV.^5^ For example, in one study^2^ the ordinal (Spearman) correlation between distal leg ENFD, an index of nerve injury, and DNP severity as measured by the VAS was −0.25, suggesting that peripheral denervation accounted for less than 10% of the variance in DNP. Correlations of DNP with other measures of nerve injury such as sural sensory nerve action potential amplitude and toe heat-pain quantitative sensory testing threshold were similarly small.^72–74^ Our findings provide potential central mechanism of dysfunctional pain relief-related processing, not dissimilar to that found in other chronic pain syndromes and as the underlying mechanism for chronification of pain.^49^^,^^75^

Limitations of the current study include its cross-sectional design, which leaves us unable to explore possible cause and effect relationships between expectation of pain relief mechanisms and HIV DNP. In addition, we examined an all-male sample which prevents generalizing our findings to the female population, which would be important to address in future work. Although our sample size (*n* = 61) is comparable with other recent studies using task-based functional MRI, future work with larger samples will be required to determine replicability of our findings in this population. The strengths of the current study include our use of painful and non-painful sites as a within-participant control for the endogenous pain in the neuropathic pain group. It is important to note that we had no detectable hands involvement in the sample in our study and thus used hand stimulation as a control condition for the foot stimulation. It is, however, possible that we could not accurately access hand sensitivity due to counterirritation in the pain group. Nevertheless, we still observed significant brain response between the two sites in our sample. Additional strengths include our use of multiple clinical measures to assess peripheral neuropathy symptoms and signs, as well as DNP and non-neuropathic pain severity. Assessment of current or state DNP and non-neuropathic pain directly before obtaining neuroimaging data also represents a strength, as there may be different brain mechanisms underlying the state and trait aspects of pain.^76^

In conclusion, differences in CNS processing of expectation of pain offset or expectation of pain relief are one possible explanation for the variation in expression of DNP for HIV. Although preliminary, our findings are in line with the motivation-decision model of pain whereby the decision is being made between avoiding pain versus seeking pain relief, the two processes that are occurring when one is faced with a pain stimulus.^9^ Neural processes mostly related to pain avoidance play a role before the pain onset, while neural processes related to expectation of pain relief play a role once the pain occurs. We found no between-group differences in the neural response to expectation of pain onset. During expectation of pain offset, our findings are consistent with the hypothesis that the pain group was hurting more due to impaired (de-conditioned) pain relief mechanisms. We believe that those with pain in our study did not see the end of pain when the pain was long, critically when the stimulus was applied to the neuropathic pain site. A similar mechanism was not seen during anticipation of pain onset in our study, as our study design did not inform participants on how long the upcoming pain was going to last, thus they did not have a significantly different anticipation of pain onset.

## Funding

This work was supported in part by the United States Department of Veterans Affairs I01-CX-000816, I01-CX- 001652, I01-CX001542, National Institute of Arthritis and Musculoskeletal and Skin Diseases of the National Institutes of Health under Award Numbers U19AR076737, K23NS079311 and Painless Research Foundation.

## Competing interests

The authors report no competing interests.

## Abbreviations

AFNI =: Analysis of Functional NeuroImages
AI =: anterior insula
BPI =: Brief Pain Inventory
DNP =: distal neuropathic pain
DSP =: distal sensory polyneuropathy
ENFD =: epidermal nerve fibre density
UC =: University of California
